# Effects of Verapamil SR and Atenolol on 24-Hour Blood Pressure and Heart Rate in Hypertension Patients with Coronary Artery Disease: An International Verapamil SR-Trandolapril Ambulatory Monitoring Substudy

**DOI:** 10.1371/journal.pone.0122726

**Published:** 2015-04-02

**Authors:** Scott J. Denardo, Yan Gong, Rhonda M. Cooper-DeHoff, Csaba Farsang, Matyas Keltai, László Szirmai, Franz H. Messerli, Anthony A. Bavry, Eileen M. Handberg, Giuseppe Mancia, Carl J. Pepine

**Affiliations:** 1 Division of Cardiovascular Medicine, College of Medicine, University of Florida, Gainesville, Florida, United States of America; 2 Center for Pharmacogenomics, College of Pharmacy, University of Florida, Gainesville, Florida, United States of America; 3 Department of Pharmacotherapy and Translational Research, College of Pharmacy, University of Florida, Gainesville, Florida, United States of America; 4 St Imre Teaching Hospital Cardiometabolic Centre, Budapest, Hungary; 5 Semmelweis University, Hungarian Institute of Cardiology, Budapest, Hungary; 6 N&Sz StudyMaster Medical Research Center Ltd., Szentendre, Hungary; 7 Division of Cardiology, St Luke’s-Roosevelt Hospital Center and Columbia University, College of Medicine and Physicians, New York, New York, United States of America; 8 North Florida/South Georgia Veterans Affairs Health System, Gainesville, Florida, United States of America; 9 Clinica Medica, Ospedale San Gerardo dei Tintori Monza, University of Milano-Bicocca, Milan, Italy; Johns Hopkins University SOM, UNITED STATES

## Abstract

Elevated nighttime blood pressure (BP) and heart rate (HR), increased BP and HR variability, and altered diurnal variations of BP and HR (nighttime dipping and morning surge) in patients with systemic hypertension are each associated with increased adverse cardiovascular events. However, there are no reports on the effect of hypertension treatment on these important hemodynamic parameters in the growing population of hypertensive patients with atherosclerotic coronary artery disease (CAD). This was a pre-specified subgroup analysis of the INternational VErapamil SR-Trandolapril STudy (INVEST), which involved 22,576 clinically stable patients aged ≥50 years with hypertension and CAD randomized to either verapamil SR- or atenolol-based hypertension treatment strategies. The subgroup consisted of 117 patients undergoing 24-hour ambulatory monitoring at baseline and after 1 year of treatment. Hourly systolic and diastolic BP (SBP and DBP) decreased after 1 year for both verapamil SR- and atenolol-based treatment strategies compared with baseline (*P*<0.0001). Atenolol also decreased hourly HR (*P*<0.0001). Both treatment strategies decreased SBP variability (weighted standard deviation: *P* = 0.012 and 0.021, respectively). Compared with verapamil SR, atenolol also increased the prevalence of BP and HR nighttime dipping among prior non-dippers (BP: OR = 3.37; 95% CI: 1.26 – 8.97; *P* = 0.015; HR: OR = 4.06; 95% CI: 1.35-12.17; *P* = 0.012) and blunted HR morning surge (+2.8 vs. +4.5 beats/min/hr; *P* = 0.019). Both verapamil SR- and especially atenolol-based strategies resulted in favorable changes in ambulatory monitoring parameters that have been previously associated with increased adverse cardiovascular events.

**Trial Registration:**

Clinicaltrials.gov; NCT00133692

## Introduction

Studies of ambulatory blood pressure (BP) and heart rate (HR) monitoring in hypertension patients have shown that abnormalities in certain hemodynamic parameters are associated with increased adverse cardiovascular events: elevated nighttime BP and HR [1–7], increased BP and HR variability [8–13], blunting of nighttime dipping [1,4,14–19], and, although somewhat controversial, augmentation of morning surge of BP and HR [19–25]. There are reports on the effect of hypertension treatment on some of these important hemodynamic parameters in the generalized hypertensive population [26–30]. However, there are no reports on any of these parameters focused on the growing population of patients with hypertension and concurrent atherosclerotic coronary artery disease (CAD).

The INternational VErapamil SR-Trandolapril STudy (INVEST) was a prospective, randomized, open label, blinded end-point study of 22,576 patients aged ≥50 years with clinically stable hypertension and CAD. INVEST compared outcomes using verapamil SR- vs. atenolol-based hypertension treatment strategies [31]. The primary outcome was the first occurrence of all-cause death, nonfatal myocardial infarction (MI), or nonfatal stroke. As previously reported [32], both strategies provided excellent office-measured BP control (>70% patients achieved office-based BP <140/90 mmHg) and were equivalent for reducing mortality and major morbidity. Here we report the results of a pre-specified detailed analysis focusing on 117 INVEST patients who underwent 24-hour ambulatory monitoring prior to randomization (“baseline”) and after 1 year of treatment to determine the effect of each treatment strategy on the above important hemodynamic parameters.

## Materials and Methods

The protocol for this trial and supporting TREND checklist are available as supporting information; see S1 TREND Checklist and S1 Protocol.

### Ethics Statement

The study was performed in accordance with the Declaration of Helsinki and was approved by the University of Florida Institutional Review Board, Gainesville, Florida (Protocol #337–2008, approved 5/22/1997). INVEST is registered at Clinicaltrials.gov, identifier NCT00133692. At the time of patient recruitment, clinicaltrials.gov was in development. The registry was made public in 2000, at which time primarily NIH-funded trials were registered. Our trial was registered in 2005 to comply with forthcoming expansion of registration requirements. The authors confirm that all ongoing and related trials for this drug/intervention are registered. All patients provided written informed consent. Patient visits occurred between 09/02/1997 and 02/14/2003.

The INVEST design, methods and principal results have been described in detail [31,32]. The study was performed in accordance with the Declaration of Helsinki and was approved by local ethics committees. All patients provided written informed consent. Briefly, patients with clinically stable hypertension and CAD were randomized to either a verapamil SR- or an atenolol-based treatment strategy. Additional nighttime dosing of the study drug and subsequent addition of trandolapril with or without hydrochlorothiazide (HCTZ) for the verapamil SR group and addition of HCTZ with or without trandolapril for the atenolol group was recommended if needed for BP control. Trandolapril was also recommended for patients with history of heart failure, diabetes, or renal insufficiency. The BP treatment goal was an office-based BP <140/90 mmHg (<130/85 mmHg for patients with diabetes and/or renal insufficiency). All adjustments in drugs were completed within 6 months of randomization.

Ambulatory monitoring was conducted in 141 INVEST patients selected by clinics with interest and expertise in ambulatory monitoring in Hungary and the United States (Meditech ABPM, Meditech Ltd., Budapest, Hungary; SpaceLabs Model 90207, SpaceLabs Medical Inc., Issaquah, WA, USA). The monitors were validated according to international protocols and measured BP and HR every 15 min from 06:00–22:00 (“daytime” hours) and every 20 min from 22:01–05:59 (“nighttime” hours). The following criteria were mandatory for inclusion into ambulatory monitoring data analysis: (1) adequate technical quality for ≥85% of the 24-hour recording period, (2) <3 consecutive hours without valid measurements, (3) <4 non-consecutive hours without valid measurements.

### Statistical Analyses

The individual BP and HR measurements for each subject were averaged into 1-hour epochs prior to subsequent analysis. Pulse pressure was defined as the difference between systolic (S) BP and diastolic (D) BP. To quantify BP and HR variability, we calculated the weighted standard deviation (wSD) and weighted coefficient of variation (wCV) [33,34].

Nighttime dipper status for SBP, DBP, and HR was defined as a decrease in SBP, DBP, or HR, respectively, by ≥10% during the hours 20:00–02:00 [18]. However, because there is no consensus for the definition of BP or HR morning surges [24,35], we calculated the difference between the minimum and maximum BP and HR, respectively, between 02:00–10:00. We also calculated the average and hourly maximum slope of the BP and HR curve between 20:00–02:00 and between 02:00–10:00.

Data for continuous variables are summarized as mean±SD or median with interquartile range, based upon symmetry of distribution. Categorical variables are presented as number (percentages). Comparisons between baseline and post–1-year treatment values of BP and HR were performed using the paired Student *t*-test (2-tailed). Independent *t* test was used for comparisons between treatment strategies. Repeated measure analysis with autoregressive 1 covariance structure was also performed to assess the difference between the 2 treatment groups and between the baseline and post–1-year visits, while adjusting for covariates including age, gender, smoking, history of MI, stroke or transient ischemic attack, and diabetes. The McNemar test was used to compare the proportion of dippers before and after treatment. Multivariable logistic regression was used to assess the predictors of dipper status and change in dipper status after adjusting for covariates. All variables with a univariate *P* value of <0.2 were considered for stepwise selection for the logistic regression model. Variables with *P*<0.05 were retained in the final model. Odds ratios and 95% confidence intervals are presented. Hosmer-Lemeshow test of goodness-of-fit was performed to evaluate the model fit. All analysis was performed using SAS version 9.3 (SAS Institute Inc, Cary, North Carolina). P-values of <0.05 were considered statistically significant.

## Results

**Fig 1:**
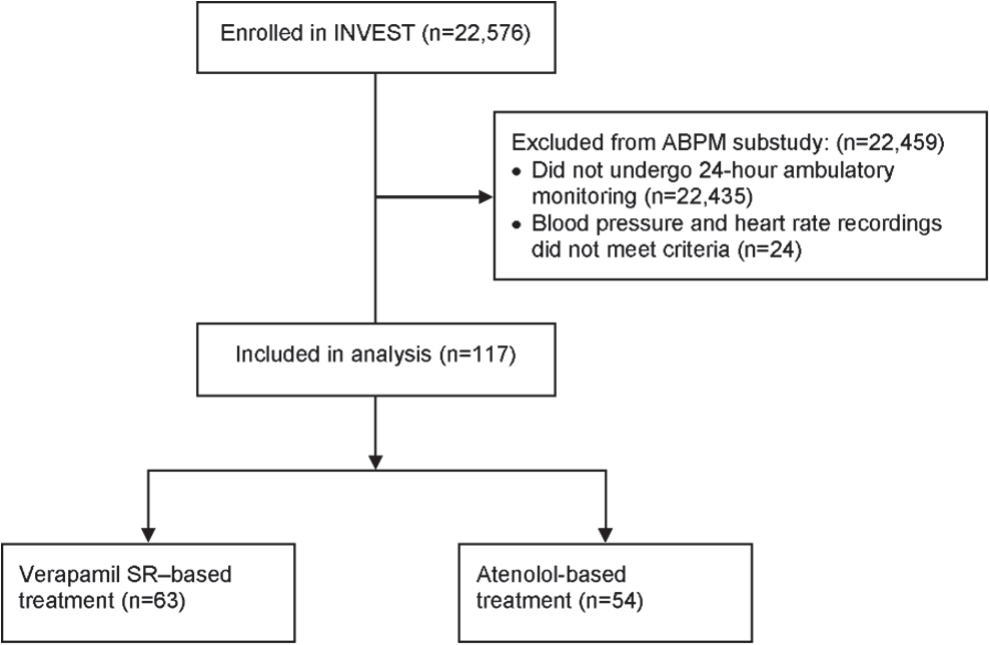
Consort diagram showing selection of INVEST patients for the ambulatory monitoring substudy analysis. The subgroup consisted of 141 patients undergoing 24-hour ambulatory monitoring at baseline and after 1 year of treatment. Patients were excluded if their blood pressure and heart rate recordings did not meet the criteria for inclusion (adequate technical quality ≥85% of the 24-hour recording period, <3 consecutive hours without valid measurements, and <4 non-consecutive hours without valid measurements).

**Fig 2:**
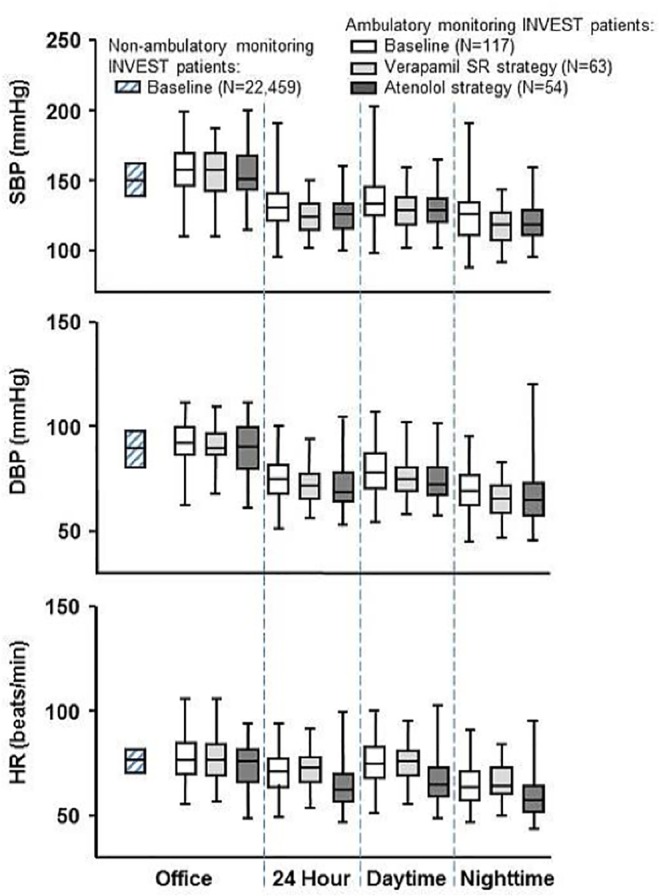
Office-based and 24-hour ambulatory monitoring systolic blood pressure (SBP), diastolic blood pressure (DBP), and heart rate (HR) at baseline and following 1 year of treatment. The baseline data contain both verapamil SR- and atenolol-based strategies combined, while the data following 1 year of treatment is individualized to treatment strategy. For comparison, baseline office-based data for the remaining INVEST patients, who did not have ambulatory blood pressure monitoring, are shown to the left. Horizontal line through each box represents median; bottom and top of box represent first and third quartiles; the whiskers represent minimum and maximum of all data.

The 141 patients with ambulatory monitoring measurements were recruited from 13 sites, representing a 68% participation rate for this substudy. Technically valid BP and HR recordings were available in 117 patients both at baseline and after 1 year of treatment (Fig 1). Baseline clinical characteristics were similar for these 117 patients comparing treatment strategies (Table 1). Additionally, baseline office SBP, DBP, and HR were similar for these patients and were also similar to the remaining 22,459 INVEST patients (Fig 2).

**Table 1:** Baseline Clinical Characteristics of Ambulatory Monitoring Patients According to Treatment Strategy, and Compared to Remaining, Non-Ambulatory Monitoring INVEST Patients.

	**Ambulatory Monitoring Substudy Patients** ^a^		**Remaining INVEST Patients**	
	**Verapamil SR-based**	**Atenolol-based**		
**Characteristic**	**(N = 63)**	**(N = 54)**	**(N = 22,459)**	**P-value** ^b^
**Age, years**	60.8 (7.5)	62.3 (7.5)	65.7 (9.8)	< 0.0001
**Women**	32 (50.8)	32 (59.3)	11706 (52.1)	0.58
**BMI, kg/m** ^**2**^	27.8 (3.8)	28.4 (4.4)	29.2 (7.1)	0.005
***History of*:**				
**Myocardial infarction**	28 (44.4)	23 (42.6)	7167 (31.9)	0.007
**Angina pectoris**	46 (73.0)	40 (74.1)	14959 (66.6)	0.11
**Coronary revascularization (CABG and/or PCI)**	3 (4.8)	7 (12.96)	6156 (27.4)	<0.0001
**Stroke/TIA**	6 (9.5)	5 (9.3)	1618 (7.2)	0.36
**LVH**	25 (39.7)	26 (48.2)	4897 (21.8)	< 0.0001
**Arrhythmia**	2 (3.2)	6 (11.1)	1592 (7.1)	0.92
**Heart failure (class I-III)**	3 (4.8)	5 (9.3)	1248 (5.6)	0.02
**Peripheral vascular disease**	4 (6.4)	2 (3.7)	2693 (12.0)	0.04
**Smoking**	24 (38.1)	19 (35.2)	10411 (46.4)	0.038
**Diabetes**	12 (19.1)	12 (22.2)	6376 (28.4)	0.059
**Hypercholesterolemia**	47 (74.6)	36 (66.7)	12510 (55.7)	0.0009
**Renal impairment**	1 (1.6)	1 (1.9)	422 (1.9)	0.89
***Antihypertensive Therapy (prior to randomization)***				
**Beta blocker** ^**c**^	0	0	0	N/A
**Calcium antagonist**	31 (49.2)	31 (57.4)	8027 (35.7)	0.0001
**Diuretic**	23 (36.5)	24 (44.4)	7346 (32.7)	0.086
**Central acting**	11 (17.5)	8 (14.8)	1033 (4.6)	<0.0001
**ACE inhibitor**	47 (74.6)	40 (74.1)	9962 (44.4)	<0.0001
**Alpha blocker**	4 (6.4)	6 (11.1)	1648 (7.3)	0.62
**Other class**	2 (3.2)	2 (3.7)	4358 (19.4)	<0.0001

Data are presented as mean (SD) or number (percent).

BMI, body mass index; CABG, coronary artery bypass graft; INVEST, INternational VErapamil SR-Trandolapril STudy; LVH, left ventricular hypertrophy; PCI, percutaneous coronary intervention; SD, standard deviation; TIA, transient ischemic attack.

^a^Comparing ambulatory monitoring study patients randomized to verapamil SR- vs. atenolol-based treatment strategies, *P* value uniformly nonsignificant.

^b^Comparing all ambulatory monitoring INVEST study patients with remaining, non-ambulatory monitoring patients.

^c^Patients taking beta-blockers within 2 weeks of randomization or taking beta-blockers for an MI that occurred in the previous 12 months were excluded from INVEST to avoid withdrawal phenomena in patients randomized to the verapamil-based treatment strategy [31].

**Fig 3:**
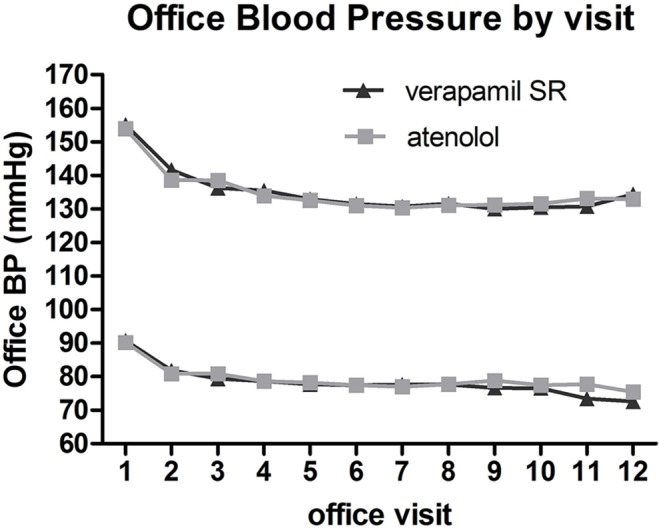
Office-based systolic and diastolic blood pressure based upon treatment strategy among 423 frequency-matched INVEST patients who did not have ambulatory blood pressure monitoring. The minimum *P* values were 0.12 and 0.09, respectively.

However, several clinical characteristics of the ambulatory monitoring patients, as a group, differed significantly from the remaining, non-ambulatory monitoring INVEST patients (Table 1). The ambulatory monitoring patients were slightly younger and less obese but had a higher prevalence of other comorbidities (e.g., MI, left ventricular hypertrophy, heart failure, and hypercholesterolemia) compared with remaining INVEST patients. To explore whether the difference in characteristics affected applicability of the subgroup analysis to the remaining INVEST patients, we created a 3:1 frequency-matched patient dataset (N = 423) for the ambulatory monitoring patients, using the remaining INVEST patients as the source. This dataset was based upon age (decades), gender, and maximized a match for the remaining 23 characteristics. The total number of characteristics successfully matched was 14/25. Using this dataset, we then compared the office BP by treatment strategy throughout the study visits spanning 48 months and found no statistical difference based on treatment strategy (all *P* values >0.05) (Fig 3). Therefore, it seems that there was no significant selection bias into the subgroup analysis and that the results of the subgroup analysis should be reasonably applicable to the remaining INVEST patients.

After 1 year of treatment, 24/63 patients randomized to the verapamil SR-based treatment strategy (38.1%) and 19/54 patients randomized to the atenolol-based treatment strategy (35.2%) had increased to a final twice-daily dosing, as directed by protocol, to optimize management of hypertension (Table 2; *P* = 0.75 for proportion). Additionally, by 6 months of treatment, 42/63 patients randomized to the verapamil SR-based treatment strategy (66.7%) had trandolapril added per protocol. However, 0/54 patients randomized to the atenolol-based treatment strategy (0%) had trandolapril added per protocol (*P*<0.0001). Finally, 20/63 (31.7%) and 33/54 (61.1%) had HCTZ added, respectively (*P* = 0.0015).

**Table 2:** Study Drug Use in Patients Randomized to Verapamil SR- or Atenolol-Based Treatment Strategy at Baseline (Immediately Following Randomization) and After 1 Year of Treatment.

**Drug**	**Baseline**		**1 Year** ^a^			
	**QD patient No/dose (median)**	**BID patient No/Dose (median)**	**QD patient No/Dose (median)**	**BID patient No/Dose (median)**	**P-value** ^b^	**P-value** ^c^
**Verapamil SR (N = 63)**	No: 61	No: 2	No: 37	No: 24	<0.0001	0.75
	Dose: 240	Dose: 360	Dose: 180	Dose: 360		
**+ Trandolapril**	No: 7	No: 2	No: 20	No: 22	<0.0001	<0.0001
	Dose: 2	Dose: 4	Dose: 2	Dose: 4		
**+ HCTZ**	No: 3	No: 0	No: 16	No: 4	0.0001	0.0015
	Dose: 25	Dose: N/A	Dose: 25	Dose: 50		
**Atenolol (N = 54)**	No: 51	No: 3	No: 32	No: 19	<0.0001	
	Dose: 50	Dose: 50	Dose: 50	Dose: 100		
**+ HCTZ**	No: 2	No: 1	No: 28	No: 5	<0.0001	
	Dose:25	Dose: 25	Dose: 25	Dose: 50		
**+ Trandolapril**	No: 6	No: 1	No: 0	No: 0	N/A	
	Dose: 2	Dose: 4	Dose: N/A	Dose: N/A		

Doses are mg/day.

N/A = not applicable.

^a^2 patients randomized to the verapamil SR strategy and 3 patients randomized to the atenolol strategy discontinued the study drug due to side effects.

^b^P-values using Wilcoxon-rank sum test comparing the doses between once daily (QD) and twice daily (BID) pts.

^c^Comparing ambulatory monitoring study patients randomized to verapamil SR- vs. atenolol-based treatment strategies for BID dosing of study drug, addition of trandolapril and addition of hydrochlorothiazide (HCTZ), per INVEST protocol.

**Fig 4:**
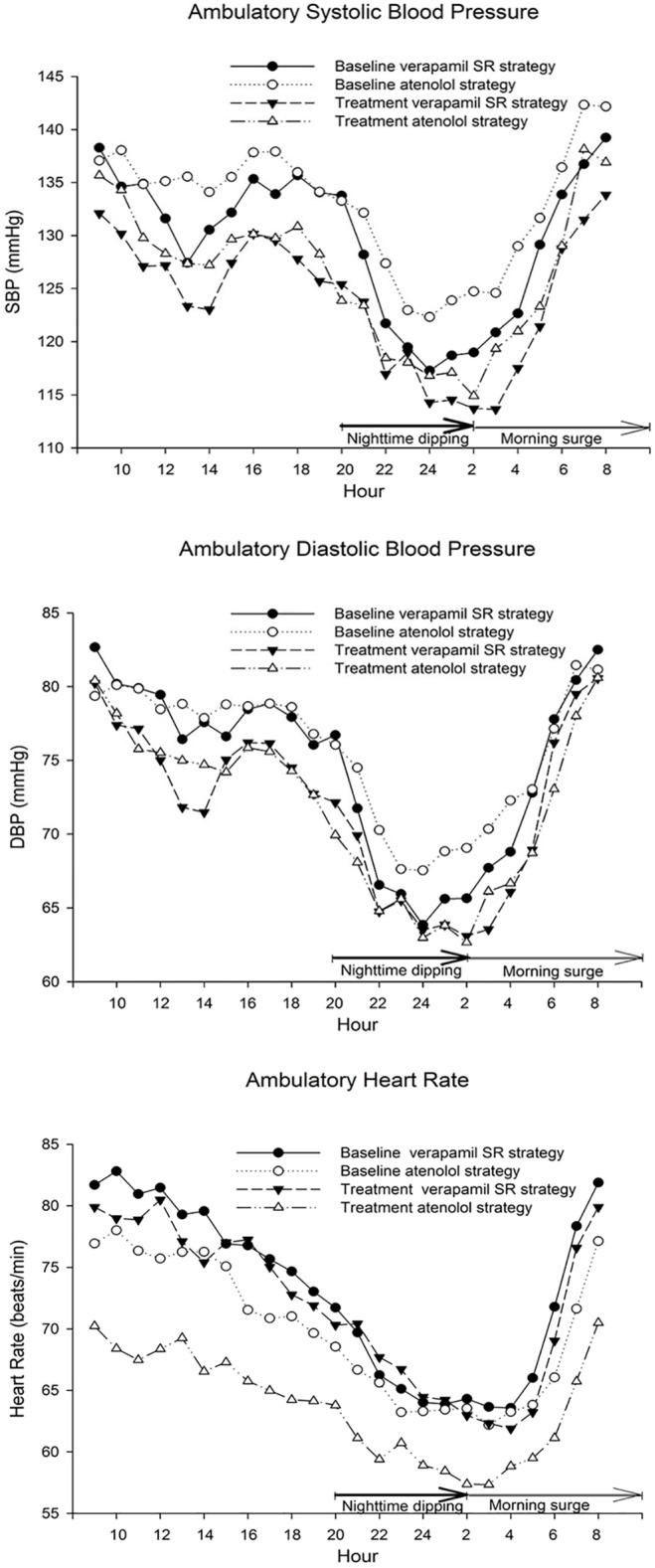
Twenty-four-hour ambulatory systolic blood pressure (SBP), diastolic blood pressure (DBP), and heart rate by strategy, both at baseline and after 1 year of treatment. Individual data points represent mean values. Nighttime dipping was determined over the time interval 20:00–02:00 and morning surge over the interval 02:00–10:00.

After 1-year of treatment, both verapamil SR- and atenolol-based strategies similarly decreased ambulatory BP vs. baseline, and this decrease persisted throughout 24 hours for each strategy (*P*<0.0001 for SBP and DBP from the repeated measure analysis; Figs 2 and 4). Additionally, there was a corresponding decrease in the 24-hour area under the BP curve for both strategies (verapamil SR: 2990/1733 vs. 2854/1673 mmHg hr, *P* = 0.011 and 0.034, respectively; atenolol: 3059/1765 vs. 2895/1691 mmHg hr, *P* = 0.0016 and 0.067, respectively). Moreover, after treatment, HR was consistently decreased among atenolol patients (*P*<0.0001 from the repeated measure analysis; Figs 2 and 4; area under HR curve: 1615 vs. 1498 beats hr/min, *P* = 0.0028) but unchanged among verapamil SR patients (*P* = 0.49; 1685 vs. 1667 beats hr/min, *P* = 0.49). Interestingly, pulse pressure at baseline was relatively low for each strategy and decreased for both after 1 year of treatment (verapamil SR: 55.5 vs. 51.8 mmHg, *P* = 0.022; atenolol: 56.6 vs. 52.5 mmHg, *P* = 0.010).

The wSD for SBP decreased with both treatment strategies (verapamil SR: 14.34 vs. 13.00 mmHg, *P* = 0.012; atenolol: 15.18 vs. 13.50 mmHg, *P* = 0.021). Also, the wCV for SBP decreased numerically—but not to statistical significance—with both strategies (11.01 vs. 10.52, *P* = 0.36; 11.46 vs. 10.76, *P* = 0.63, respectively). Conversely, the wSD and wCV for DBP and HR did not change with either treatment strategy.

At baseline, 68% and 56% of the patients were BP dippers within the verapamil-SR and the atenolol strategies, respectively (*P* = 0.14, comparing strategies), while 65% and 69% of the patients were BP dippers after treatment, respectively (*P* = 0.61, comparing strategies). However, 29.6% of atenolol BP non-dipper patients vs. 11.1% of verapamil SR BP non-dipper patients changed to dippers after 1-year (*P* = 0.028), and 25.9% vs. 7.9% changed from HR non-dipper to dipper, respectively (*P* = 0.0007). Additionally, the average slope of the nighttime SBP curve was less steep (i.e., blunting of nighttime SBP dipping) for the verapamil SR patients after 1 year of treatment compared with baseline (-2.1 vs. -4.3 mmHg/hr, respectively; *P* = 0.026) (Fig 4). However, after treatment, there was no significant change in the average slope of the nighttime SBP curve for the atenolol patients, no significant change in the average slope of the nighttime DBP curve for either drug, and no significant changes in maximum slopes for either drug for either nighttime SBP or DBP curves (Fig 4). Finally, there was no significant change after treatment in the average or maximum slopes of the nighttime HR curve for either drug (Fig 4).

Logistic regression showed that BP dipper status at baseline strongly predicted 1-year treatment dipper status; patients who were dippers at baseline were almost 3 times as likely to be a dipper after 1 year of treatment, compared to non-dippers (OR: 2.93; 95% CI: 1.30–6.57; *P* = 0.0094). For HR dipper status, baseline dipper status was marginally significant (OR: 2.11; 0.88–5.06; *P* = 0.094). Treatment with the atenolol-based strategy was a significant predictor for changing from a BP non-dipper to a dipper (OR: 3.37; 95% CI: 1.26–8.97; *P* = 0.015) and HR non-dipper to a dipper (OR: 4.06; 95% CI: 1.35–12.17; *P* = 0.012). Interestingly, baseline demographics such as age, gender, history of MI, heart failure, and diabetes did not predict changes in dipper status. The *P* values for Hosmer-Lemeshow test of goodness-of-fit were uniformly >0.05.

There were no significant differences, augmentation, or blunting of BP morning surge comparing strategies either at baseline or at 1 year (Fig 4). All BP slopes were similar. However, there was blunting of HR morning surge for atenolol vs. verapamil SR patients at 1 year (Fig 4; +2.8 vs. +4.5 beats/min/hr, respectively; *P* = 0.019).

## Discussion

The results of this substudy of the INVEST indicate that, for patients with hypertension and CAD, both verapamil SR- and atenolol-based treatment strategies provide 24-hour BP control with positive effects on important hemodynamic parameters that have been previously associated with adverse cardiovascular events. These positive effects may have contributed to limiting adverse events and include: (1) a consistent decrease in nighttime BP; (2) a decrease in 24-hour SBP variability; and (3) no overt negative effect on diurnal BP variations. Additionally, atenolol provided a consistent decrease in nighttime HR, a relative increase in change from non-dipper to dipper status for both BP and HR, and a blunting of HR morning surge. These latter effects may have contributed to the limitation in adverse events observed among congestive heart failure patients receiving the atenolol-based treatment strategy in the INVEST [32].

The ambulatory monitoring used in this substudy provided a more continuous measurement of BP and HR for these patients over 24 hours compared with the remaining INVEST patients, who had office BP and HR measured at one time point during scheduled visits. Ambulatory monitoring in clinical trials has been shown to provide enhanced precision (allowing for reduced sample size and/or increased study power), elimination of observer bias, and identification of individuals with “white coat,” “masked” and even true “treatment resistant” hypertension [36]. Moreover, ambulatory monitoring before the start of lifelong drug treatment may lead to more appropriate targeting of treatment, particularly around the diagnostic threshold [37].

Unfortunately, the recently published JNC8 Report [38] did not address the use of ambulatory monitoring for BP management, and similarly did not specifically address patients with hypertension and CAD as a special population. Thus the 24-hour effects of antihypertensive drugs on the growing population of patients with hypertension and CAD—including issues such as "masked hypertension,” true "treatment resistant hypertension," and the concept of optimizing the target of treatment around the diagnostic threshold—are not addressed by our most current national hypertension management guidelines.

This substudy has limitations. First, patients undergoing ambulatory monitoring represented a pre-specified population of interest. However, they were selected by clinics in Hungary and the United States with interest and expertise in ambulatory monitoring. Additionally, the patients were not randomly selected and demonstrated some clinical characteristics that differed from the remaining INVEST patients. Nonetheless, their similarity in remaining baseline characteristics, baseline BP and HR and the results of our frequency-matched patient dataset analysis do suggest that the results of the subgroup analysis are reasonably applicable to the remaining INVEST patients. Second, day-night blood pressure changes and the classification of patients into dippers and non-dippers can be poorly reproducible over time [39], which can limit the applicability of those results. Third, the effect of other non-randomized antihypertensive drugs used in the INVEST (e.g., trandolapril and HCTZ) may have a confounding effect on the results. Finally, the relatively low baseline pulse pressure and its subsequent decrease for both strategies may have independently contributed to limiting adverse events in all INVEST patients.

Limitations notwithstanding, the results of this substudy of INVEST using ambulatory monitoring demonstrate that both verapamil SR- and especially atenolol-based strategies result in favorable changes in ambulatory monitoring parameters for patients with hypertension and CAD that have been previously associated with increased adverse cardiovascular events.

## Supporting Information

S1 TREND Checklist TREND Checklist. (PDF)

S1 Protocol Comparison of Non-Invasive Blood Pressure Methodologies: A Substudy of the International Verapamil SR/Trandolapril Study (INVEST). (PDF)
