# Correction: Effects of Verapamil SR and Atenolol on 24-Hour Blood Pressure and Heart Rate in Hypertension Patients with Coronary Artery Disease: An International Verapamil SR-Trandolapril Ambulatory Monitoring Substudy

**DOI:** 10.1371/journal.pone.0157212

**Published:** 2016-06-03

**Authors:** Scott J. Denardo, Yan Gong, Rhonda M. Cooper-DeHoff, Csaba Farsang, Matyas Keltai, László Szirmai, Franz H. Messerli, Anthony A. Bavry, Eileen M. Handberg, Giuseppe Mancia, Carl J. Pepine

There is an error in the affiliation for the tenth author, Giuseppe Mancia. Affiliation #9 should be:

University of Milano Bicocca and IRCCS Istituto Auxologico Italiano, Milan, Italy.

## References

[1] Denardo SJ, Gong Y, Cooper-DeHoff RM, Farsang C, Keltai M, Szirmai L, et al (2015) Effects of Verapamil SR and Atenolol on 24-Hour Blood Pressure and Heart Rate in Hypertension Patients with Coronary Artery Disease: An International Verapamil SR-Trandolapril Ambulatory Monitoring Substudy. PLoS ONE 10(4): e0122726 doi:10.1371/journal.pone.0122726 25835002 10.1371/journal.pone.0122726 PMC4383326

