# Supplementary material for: Effects of Verapamil SR and Atenolol on 24-Hour Blood Pressure and Heart Rate in Hypertension Patients with Coronary Artery Disease: An International Verapamil SR-Trandolapril Ambulatory Monitoring Substudy
Source: PLoS One. 2015 Apr 2;10(4):e0122726. doi: 10.1371/journal.pone.0122726 (PMC4383326; doi:10.1371/journal.pone.0122726)
Supplement: S1 Protocol — (PDF) [file pone.0122726.s002.pdf]

Protocol

1. Project Title:

Comparison of Non-Invasive Blood Pressure Methodologies: A Substudy of The International Verapamil SR/Trandolapril Study (INVEST).

2. Investigator(s):

Eileen Handberg, PhD, ARNP
Patrick Heyman, MSN
Carolyn Yucha, PhD
Carl J. Pepine, M.D.
Rhonda Cooper-DeHoff, PharmD
Julie Johnson, PharmD
Betsy Babcock, M.D.
Richard Kerensky, M.D.
Juan Aranda, M.D.
Hal Brodsky, M.D.
David Sheps, M.D.
Karen Hall, M.D.

3. Abstract:

The diagnosis and treatment of hypertension has traditionally been based on office blood pressure measurements, although it is well-known that a single office reading is influenced by several factors, like emotional state, time of the day, physical activity (1,2). It is also well established, that office blood pressure readings overestimate the patient's average blood pressure outside the medical setting (3). Since the availability of accurate, small and inexpensive ambulatory blood pressure monitors improved dramatically in the 1990's, several studies were performed to examine the predictive value of such devices in the determination of the risk of cardiovascular morbidity in patients with hypertension. It has been found, that ambulatory blood pressure is an independent predictor of prognosis in essential hypertension (4), and more valuable in prognosticating the cardiovascular risk (5) and treatment-induced regression of left ventricular hypertrophy in hypertensive patients (6). Other non-invasive measurements such as applanation tonometry which measures aortic pulse pressure, augmentation index and pulse wave velocity have also been shown to provide measures of arterial compliance which were determinants of coronary artery disease severity. The rationale of this substudy is correlate office blood pressure measurements, applanation tonometry and ABPM measurements in patients participating in the INVEST. Patients will have ABPM and applanation tonometry measured at the beginning of the substudy and ABPM at the time of the closeout visit for INVEST.

4. Specific Aims:

- To determine the correlation between the office measurements and the ABPM measurements in patients participating in the INVEST.

- To determine the circadian systolic and diastolic blood pressure parameters of patients at ABPM substudy entry and at INVEST closeout.

- To compare the two treatment strategies in INVEST in terms of differences between daytime and nighttime blood pressure values and blood pressure variability.

- Determine whether there are differences between brachial blood pressures and aortic blood pressures in INVEST participants.

- Determine whether there are differences between 24-hour average ambulatory blood pressures and aortic blood pressures in INVEST participants.

- Determine whether there are differences in the above measurements, if any, between patients who are being treated with calcium antagonist versus non-calcium antagonist based therapy.

- Characterize the augmentation index and pulse wave velocity of patients participating in INVEST.

- Determine the difference between peak and trough aortic blood pressure measurements, augmentation index, and pulse wave velocity for patients taking once daily dosing medications.

- To combine the appropriately selected Ambulatory BP measurements obtained as part of the INVEST Hungarian ABPM Substudy with this study and to compare the ABPM readings with office blood pressure.

The research protocol proposed would collect the following additional data: 1) carotid to femoral aortic pulse wave velocity as an index of arterial wall stiffness, 2) noninvasive brachial blood pressures 3) noninvasively assessed aortic blood pressure derived from radial and carotid pressure using a generalized transfer function, and 4) observed modifications of central systolic and pulse pressure determined by wave reflection (augmentation index).

5. Background and Significance:

Blood pressure measurements are among the most common measures in clinical care and physiological research. Blood pressure measurements yield a variety of parameters such as systolic, diastolic, and pulse pressure. Additionally, some methods record the contour of the pulse wave generating even more parameters such as augmentation index, time under ejection, and pulse wave velocity. With all the information that can be obtained from a given blood pressure measurement, the following questions concerning the relationship of these parameters to actual clinical outcomes should be considered: 1) Which parameter is most

important in the prediction of mortality and morbidity? 2) Which parameter should antihypertensive therapies target?

Hypertension is a major risk factor for atherosclerosis classically attributed to reduction in caliber and/or number (vascular rarefaction) of small arteries and arterioles resulting in increased peripheral resistance. Peripheral resistance (pressure drop divided by cardiac output), however, does not account for fluctuation of pressure and flow during the cardiac cycle, with systolic and diastolic pressure representing the extremes of pulse pressure fluctuations. Diastolic pressure is closer to mean pressure (and therefore to resistance) than systolic pressure, and as such has been used as a marker for the diagnosis and treatment of hypertension. However, this approach is arbitrary and was challenged by the Framingham Heart Study which demonstrated that systolic and pulse pressures rather than diastolic pressure are better markers for stroke and coronary heart disease risk (2,5). This view has been confirmed by epidemiological and intervention studies (3,4,6). Systolic pressure is closely associated with pulse pressure and is determined by LV ejection, arterial stiffness and timing of arterial wave reflection (i.e., geometrical and viscoelastic properties of large conduit arteries) (7,8,9). In humans, aging and hypertension stiffen arteries (compliance decreases) as a result of progressive degeneration of the arterial media, decreased elastin, increased collagen and calcium content and hypertrophied vascular smooth muscle in large elastic arteries (7). Thus, the increase in systolic pressure (as a result of arterial damage) accelerates arterial damage, producing a self-perpetuating cycle.

While increase in mean arterial pressure has been the hallmark of hypertension, it is not the only cause of the associated cardiac and vascular complications. Such complications are most closely related to elevation in systolic and pulse pressures (4,5,6) as a result of increased arterial stiffness and PWV and early return of reflected waves during LV systole (7,8,9). The SHEP, Syst-Eur and Syst-China studies confirm that reduction of systolic and pulse pressure, even when diastolic pressure is normal, reduces cardiovascular events (10,11,12).

Recent findings have changed the focus of attention in hypertension from blood pressure and resistive arterial properties to mechanical and functional properties of blood vessels. Characterization of the mechanisms by which arterial compliance and wave reflection modify systolic and pulse pressure and interact after treatment with antihypertensive therapy is essential to an improved understanding of these mechanical properties. Such attention should contribute to improved therapy of established hypertension.

Previous theory focused on peripheral resistance as the primary cause of hypertension and increased myocardial load. Traditionally, it has been thought and widely accepted that diastolic blood pressure correlated most closely with end organ damage because diastolic pressure was the key indicator of successful reductions in peripheral resistance (13). However recent findings suggest that pulse pressure and systolic blood pressure are more important indicators of disease in the elderly (14). In fact, very low diastolic pressures have been associated with increased coronary risk (13).

Traditional hypertension studies have used cuff-based brachial blood pressure measurements to assess the outcomes of hypertension management in clinical trials. There is mounting evidence that 24-hour average pressure measurements are more highly correlated with organ damage and regression of damage under treatment (15). Evidence also shows that aortic blood pressure measurements and arterial compliance are better indicators of clinical outcomes (13).

Peripheral measurements of blood pressure are often used because they are more convenient. However, peripheral arteries inherently have a higher pressure than do central arteries. Arteries have a tendency to stiffen over time, decreasing or increasing the peripheral/central pressure gradient (16). Because of the changing gradient with age, central pressures are more closely related to aortic elastance, a predictor of mortality (17). Accordingly, central pressures have greater relation to heart disease than peripheral measurements (18) and are more useful in the assessment of cardiovascular risk.

Conventional noninvasive blood pressure measurement as recommended by the JNC-VI (19) uses a Riva-Rocci based inflatable cuff pressure taken at the brachial artery—a muscular artery. The aorta is an elastic artery that stiffens with age (16) consequently, there can be significant differences between brachial and aortic pressures.

Newer theories also take into consideration elastance (arterial stiffness) and wave reflections (13). Cuff based measurements can only provide systolic and diastolic pressures, although mean and pulse pressure can be derived. Applanation tonometry is a noninvasive method of determining aortic pressure. Aortic pressure waveforms may be approximated by use of a transfer function from radial or carotid readings (20,21,22,23). Because organ damage is more closely associated with central pressure than peripheral (brachial) pressure, applanation tonometry allows the researcher and the clinician to obtain a clearer picture of the disease process and management than the cuff-based measurement recommended by the JNC VI (19). Applanation tonometry can also measure pulse wave velocity and augmentation index, which are a measure of arterial stiffness and wave reflections respectively.

The HOPE study (Ramipril) showed that reductions in blood pressure of only 2-3 mmHg dramatically reduced mortality rate (24,25). Traditional theory cannot explain this degree of improvement based on blood pressure lowering affects alone, elastance and wave reflection improvements resulting from pharmacological therapy might be able to. However, the study used cuff-based measurements and did not measure augmentation index or pulse wave velocity, so valuable data were lost.

6. Research Plan:

Inclusion Criteria:

1. Patients with documented hypertension, and coronary artery disease who are participating in the INVEST Trial.
2. Patient data from patients recruited in the locally approved INVEST Hungarian ABPM Substudy.

Exclusion Criteria;

1. Unwilling to provide written informed consent
2. Atrial Fibrillation
3. Unable to wear blood pressure monitoring device
4. Severe muscle tremors (e.g. Parkinsons)

Patients in the southeast currently enrolled in the INVEST trial will be approached for possible participation. If patients are agreeable and provide written informed consent, they

will have in addition to their routine office assessments of blood pressure, an ambulatory blood pressure monitor placed which will be worn for 24 hours. Blood pressure and heart rate measurements will be taken by oscillometric method uniformly every 20 minutes during a 24 hour period. Patients will be provided the monitors in the morning after the determination of the office blood pressure and heart rate. Patients will be asked to record daily activities in a diary. They will be instructed to spend the day like an "average day". The patients will be asked to return the clinic or office at the end of the 24 hour period to return the monitor. Patients will undergo evaluation of the carotid artery in the neck, the femoral artery in the leg and the radial artery in the wrist using a non-invasive method. A handheld wand (PWV/Millar pressure tonometer) connected to a measuring device (Sphygmocor Pulse Wave Analysis System) will be used to gently detect and measure the pulses in those arteries non-invasively. The wand will be placed gently on the skin of the neck, leg and wrist to measure the pressures there. The wand will remain in place for 1-2 minutes in each area. These measurements will be obtained shortly after taking the blood pressure with a standard blood pressure cuff. These measurements will be repeated at the end of the INVEST study at the time of the final visit. The non-invasive pulse and blood pressure measurement will take between 20 and 30 minutes to obtain.

Additional data will be received from the Hungarian sites. The protocol was reviewed and approved by their local ethics boards. The protocol design was to obtain baseline and 1 year ABPM measurements on INVEST patients who were recruited and consented for the Substudy. They did not collect tonometry readings.

Data Measurements for Applanation Tonometry

Aortic Pulse Wave Velocity (PWV): Pulse wave velocity between the carotid and femoral arteries will be measured and used as an index of aortic elastance (inverse of compliance). High fidelity pressure waves will be recorded non-invasively by applanation tonometry using a Millar pressure transducer and a SphygmoCor system. Pulse wave velocity will be calculated as the ratio of the distance between measuring sites and travel time of the two pressure waves using the ECG as a reference and the upstroke of the two pressure waves (7). Ten continuous beats are ensemble averaged and used by the SphygmoCor system to calculate PWV.

Amplitude (Augmentation Index) and Timing of the Reflected Pressure Wave: Augmentation index (AIc), the index of wave reflection amplitude, and travel time of the reflected wave from the periphery will be obtained from the carotid pulse recorded by applanation tonometry. Applanation index will be calculated as the ratio of amplitude of the pressure wave above its systolic shoulder to the pulse pressure (7,8,9). Travel time of the reflected wave (tp/2) from the periphery will be obtained as the time from the upstroke of the initial pressure wave to the beginning of the reflected wave (inflection point) divided by two (7,8). Pressure waves will be recorded over an eight-second period and ensemble averaged into a single wave and AIc and tp/2 calculated using the SphygmoCor system.

Cuff Blood Pressure:

Brachial cuff pressure will be measured automatically with a Dynamap system based on the guidelines provided in JNC-VI. Briefly this will require the patient to rest for 5-10 minutes and have two blood pressure measurements taken.

Determination of the Aortic Pressure Wave from the Radial Pressure Wave: Previous published results (7,26) suggest that accurate contours of the ascending aortic pressure waveform can be obtained from the radial artery pressure waveform using a generalized mathematical transfer function. In the proposed study, radial artery pressure waveforms will be recorded by applanation tonometry and central aortic pressure waveforms calculated using the SphygmoCor system. This system averages ten pressure pulses and generate indices of ventricular/vascular coupling, including ascending aortic pressure wave augmentation index, wave reflection travel time, systolic pressure time index and subendocardial viability ratio.

These measurements will be collected at regularly scheduled clinic visits over the next 6 months and then at INVEST study closeout (4th quarter 2002,1st quarter 2003).

Sample Size:

Enrollment will consist of at least 250 hypertensive patients (125 per treatment group). A two-sided test performed at an alpha-level of 5% for the correlation between the mean diastolic blood pressure measured by the ABPM device and the mean diastolic blood pressure measured in the office will then have 90% power to detect a correlation of at least 0.240. This number accounts for 10% dropouts. The demographic characteristics of the patients are anticipated to be representative of the INVEST population.

A power analysis was done using the computer software SamplePower (27) to determine the number of subjects needed to detect a 5 mmHg difference in aortic pressure between the two treatment groups using a t-test. Five mmHg was chosen, as this is the minimum difference that may be viewed to be of clinical importance. In another study conducted in our laboratory 28, the standard deviation of the aortic blood pressure measurements was approximately 10 mmHg. Using these figures, 80 subjects per group are needed to detect a clinical difference of 5 mm Hg with 95% confidence and 80% power. With 500 potential subjects in the Gainesville area, this study only needs 32% agreement to participate to have sufficient power to accurately answer the research question.

Anonymized data will be transmitted on 140 patients recruited in Hungary.

7. **Potential Health Risks:**

No health risks are present as a result of the non-invasive pulse wave and blood pressure measurements. Intermittent inflation of the blood pressure cuff during the testing may interfere with some activities of your daily life and sleep.
If you wish to discuss the information above or any other discomforts you may experience, you may ask questions now or call the Principal Investigator listed on the front page of this form.

8. **Potential Health Benefits:**

No direct health benefits are anticipated as a result of the blood pressure and pulse wave measurements. The measurement of blood pressure over a 24 hour period may provide additional information about how well a patient's blood pressure is currently controlled.

9. Potential Financial Risks:

There is no financial risk for participating in this project.

10. Potential Financial Benefits:

There is no financial benefit for the patient participating in this research.

11. Conflict of Interest:

There is no potential benefit to the investigator(s) beyond the professional benefit from academic publication or presentation of the results.

References

1 Bramwell, J.C. and Hill, A. V. (1922). Velocity transmission of the pulse wave and elasticity of arteries. Lancet, 1, 891-892.

2 Mackenzie, J. (1918). Diseases of the Heart, 3rd edition. London: Frowde, Hodder, Stoughton.

3 Kannel, W.B, Wolf, PA., McGee, D.L., Dawber, T.R., McNamara, P and Castelli, W. P (1981). Systolic blood pressure, arterial rigidity and risk of stroke. The Framingham Study. JAMA, 245, 1225-1229.

4 Rutan, G.H., Kuller, L.H., Neaton, J.D., Wentworth, D.N., McDonald, R.H. and McFate-Smith, W. (1988). Mortality associated with diastolic hypertension and isolated systolic hypertension among men screened for Multiple Risk Factor Interventional Trial. Circulation, 77, 505-514.

5 Nielsen WB, Lindenstrom E, Vestbo J, Jensen GB. (1997). Is diastolic hypertension an independent risk factor for stroke in the presence of normal systolic blood pressure in the middle-aged and elderly? American Journal of Hypertension, 10,634-639.

6 Franklin SS, Khan SA, Wong ND, Larson MG, Levy D. (199). Is pulse pressure useful in predicting risk for coronary heart disease? Circulation, 100,354-360.

7 Benetos A, Zureik M, Morcer J, Thomas F, Bean K,Safar M, Ducimetiere P, Guize L. (2000). A decrease in diastolic blood pressure combined with an increase in systolic blood pressure is associated with a higher cardiovascular mortality in men. J Am Coll Cardiol, 35:673-80.

8 Nichols, W.W. and O'Rourke, M.F. (1990). McDonalds Blood Flow in Arteries, 3 rd edition. London, Arnold.

9 Nichols WW, Avolio AP, Kelly RP, O’Rourke MF. (1993). Effects of age and hypertension on wave travel and reflections. In Arterial Vasodilation: Mechanisms and Therapy. MF O'Rourke, ME Safar, V Dzau, editors, London: Arnold.

10 O'Rourke, M.F. (1989). Systolic blood pressure, arterial compliance and early wave reflection, and their modification by antihypertensive therapy. J Hum Hyper, 3, 47-52.

11 SHEP Cooperative Research Group. (1991). Prevention of' stroke by antihypertensive drug treatment in older persons with isolated systolic hypertension: Final results of the Systolic Hypertension in the Elderly Program (SHEP).JAMA, 265, 3255-3264.

12 Liu L, Wang WG, Gong L, Staessen JA, for the Systolic Hypertension in China (Syst-China) Collaborative Group. (1998). Comparison of active treatment and placebo for older patients with isolated systolic hypertension. J Hypertens, 16:1823-1829

13 Nichols, W. W. & Edwards, D. G. (2001). Arterial elastance and wave reflection augmentation of systolic blood pressure: deleterious effects and implications for therapy. Journal of Cardiovascular Pharmacolgical Therapeutics, 6(1), 5-21.

14 Swales, J.D. (2000) Systolic versus diastolic pressure: paradigm shift or cycle? Journal of Human Hypertension, 14(8), 477-9.

15 Mancia, G., & Parati, G. (2000). Ambulatory blood pressure monitoring and organ damage. Hypertension, 36(5), 894-900.

16 Nichols, W.W., O'Rourke, M.F., Avolio, A.P., Yaginuma, T. Murgo, J.P., Pepine, C.J., & Conti, C.R. (1985). Effects of age on ventricular-vascular coupling. American Journal of Cardiology, 55(9), 1179-84.

17 Laurent, S., Boutouyrie, P., Asmar, R., Gautier, I., Laloux, B., Guize, L., Ducimetiere, P., & Benetos, A. (2000). Aortic stiffness is an independent predictor of all-cause mortality in hypertensive patients. Journal of Hypertension, 18, S20.

18 Waddell, T. K., Dart, A. M., Medley, T. L., Cameron, J. D., & Kingwell, B. A. (2001). Carotid pressure is a better predictor of coronary artery disease severity than brachial pressure. Hypertension, 38(4), 927-931.

19 JNC-VI. (1997). Sixth report of the joint national committee on the prevention, detection, evaluation and treatment of high blood pressure (JNC VI). Washington, DC: Government Printing Office, NIH Publication No. 98-4080. [On-line]. Available: http://www.nhlbi.nih.gov/guidelines/hypertension/jncintro.htm

20 Karamanoglu, M., O'Rourke, M. F., Avolio, A. P., & Kelly, R. P. (1993) An analysis of the relationship between central aortic and peripheral upper limb blood pressure waves in men. European Heart Journal, 14(2), 160-167.

21 Nichols, W. W., & O'Rourke, M. F. (1998). McDonald's blood flow in arteries: Theoretical, experimental and clinical principles (4th ed.). New York: Oxford University Press.

22 O'Rourke, M. F., & Gallagher, D. E. (1996). Pulse wave analysis. Journal of Hypertension, 14(Suppl. 5), S147-S157.

23 Siebenhofer, A., Kemp, C., Sutton, A., & Williams, B. (1999). The reproducibility of central aortic blood pressure measurements in healthy subjects using applanation tonometry and sphygmocardiography. Journal Human Hypertension, 13(9), 625-629.

24 Dagenais, G. R., Yusuf, S., Bourassa, M. G., Yi, Q., Bosch, J., Lonn, E. M., Kouz, S., & Grover, J. (2001). Effects of ramipril on coronary events in high-risk persons: results of the Heart Outcomes Prevention Evaluation Study. Circulation, 104(5), 522-526.

25 Mancini G.B., & Stewart D.J. (2001). Why were the results of the Heart Outcomes Prevention Evaluation (HOPE) trial so astounding? Canadian Journal of Cardiololgy, (Suppl. A),15A-7A.

26 Chen CH, Nevo E, Ferics B, Pak PH, Yin FCP, Maughan L, Kass DA. (1997). Estimation of central aortic pressure waveform by mathematical transformation of radial tonometry pressure; validation of generalized transfer function. Circ, 95:1827-1836.

27 Borenstein, M., Rothstein, H., & Cohen, J. (2001). SamplePower (Version 1.0) [Computer Sofware]. Chicago, IL: SPSS, Inc.

28 Tsai, P. (2001). Hemodynamics and arterial properties underlying pressure responses to cognitive stress in borderline hypertensives. (Doctoral Dissertation, University of Florida, 2001). [Online]. Available: http://etd.fcla.edu/etd/uf/2001/anp4007/PT-ETDrev.pdf.
